# Estimating Biomechanical Time-Series with Wearable Sensors: A Systematic Review of Machine Learning Techniques

**DOI:** 10.3390/s19235227

**Published:** 2019-11-28

**Authors:** Reed D. Gurchiek, Nick Cheney, Ryan S. McGinnis

**Affiliations:** 1 M-Sense Research Group, University of Vermont, Burlington, VT 05405, USA; reed.gurchiek@uvm.edu; 2 Dept. of Computer Science, University of Vermont, Burlington, VT 05405, USA; ncheney@uvm.edu

**Keywords:** machine learning, hybrid estimation, wearable sensors, electromyography, inertial sensor, regression, remote patient monitoring, joint mechanics

## Abstract

Wearable sensors have the potential to enable comprehensive patient characterization and optimized clinical intervention. Critical to realizing this vision is accurate estimation of biomechanical time-series in daily-life, including joint, segment, and muscle kinetics and kinematics, from wearable sensor data. The use of physical models for estimation of these quantities often requires many wearable devices making practical implementation more difficult. However, regression techniques may provide a viable alternative by allowing the use of a reduced number of sensors for estimating biomechanical time-series. Herein, we review 46 articles that used regression algorithms to estimate joint, segment, and muscle kinematics and kinetics. We present a high-level comparison of the many different techniques identified and discuss the implications of our findings concerning practical implementation and further improving estimation accuracy. In particular, we found that several studies report the incorporation of domain knowledge often yielded superior performance. Further, most models were trained on small datasets in which case nonparametric regression often performed best. No models were open-sourced, and most were subject-specific and not validated on impaired populations. Future research should focus on developing open-source algorithms using complementary physics-based and machine learning techniques that are validated in clinically impaired populations. This approach may further improve estimation performance and reduce barriers to clinical adoption.

## 1. Introduction

Since the turn of the century, wearable sensors have experienced substantial technological advancements that have reduced their size and power requirements, improved their wearability, and increased the quality and types of data they capture. These improvements have allowed the application of wearable sensors to important clinical challenges impacting human health. These challenges include the development of novel digital biomarkers [1] that could be used for diagnosis, prognosis, and clinical decision making in a variety of neurological [2,3], mental health [4,5], and musculoskeletal [6,7,8,9] disorders.

In many cases, clinical evaluation using these biomarkers could be enhanced by also considering remote observation made during a patient’s daily life (e.g., daily biomechanical variability is clinically informative in persons with multiple sclerosis [2]). Recent research suggests remote observations may differ than those made in the lab or clinic [10,11,12], and thus may provide additional information for informing clinical decision making. Additionally, remote observation could be used as an endpoint for assessing efficacy of interventions designed to target specific biomechanical indices (e.g., using biofeedback to reduce knee loading [13]). Taken together, these developments suggest that remote observation of patient biomechanics during daily life is emerging as an important tool for improving human health. Thanks to recent technological advancements, wearable sensors are ideally positioned to enable remote patient monitoring. However, wearable sensors do not necessarily provide direct measurement of the mechanisms underlying any particular clinical condition. Previous research on the mechanistic origins of various diseases (e.g., musculoskeletal [14,15,16], neurological [17]) motivate the incorporation of physically interpretable biomarkers as a part of a comprehensive patient evaluation. These biomarkers, when observed continuously via remote patient monitoring, may then directly inform an optimal clinical intervention [18,19,20]. In this review we focus on the estimation of physically interpretable biomarkers for musculoskeletal and neurological disorders which take the form of biomechanical time-series representing joint, segment, and muscle kinetics and kinematics.

### 1.1. Physical Models

The aforementioned biomechanical time-series may be determined from wearable sensor data using established mathematical relationships governed by physical models. For example, strapdown integration [21] of the angular rate signal from a segment attached gyroscope is a physics-based estimate of segment orientation where an accompanying accelerometer and magnetometer may provide the initial conditions and drift correction over time (e.g., see [6]). The development of sensor fusion techniques for removing integration drift in orientation estimates has been (and continues to be) a research focus [21,22]. Inertial sensor estimates of segment kinematics are sufficient to estimate joint kinetics during open-chain tasks using an inverse-dynamics approach given estimates of segment inertial and geometric parameters [23]. However, additional sensors are needed for closed-kinetic chain tasks since then external contact forces must be considered (i.e., measured). Alternatively, wearable surface electromyography (sEMG) sensors may inform a solution for the net joint moment using Hill-type muscle models and thus also joint and/or segment kinematics for open-chain tasks via forward-dynamics [24,25,26]. However, as noted in [27], it is quickly realized that the number of sensors required to inform a physical model is inhibitive since the muscle activation of every muscle must be estimated thus limiting the use of these approaches for remote patient monitoring.

One solution is to simplify the physical model such that a reduced number of sensors can be used to measure all required independent variables. Many techniques for simplification have been proposed and are context dependent. For example, sacral accelerations have been assumed to represent those of the center of mass enabling a single inertial sensor estimate of ground reaction force [28]. For muscle force estimation, muscle contraction dynamics are often simplified to comply with a lumped-parameter Hill-type model as opposed to a continuum model [29,30,31,32]. Further, it is common practice to assume unobserved muscle states (e.g., activation, tension) can be computed in terms of a single or multiple synergistic muscles whose states are available (e.g., via sEMG) [24,27,33]. Recently, Dorschky et al. (2019) present a physics-based technique for estimation wherein the states of a neuromusculoskeletal model (including the biomechanical time-series of interest) were optimized to agree with measured sensor data using trajectory optimization [34]. While the results were promising, the model was only two-dimensional, requires an inertial sensor on each of seven segments, and was further limited by computation time (mean CPU time was 50 ± 26 min across 60 optimizations where each optimization had 10 strides). The model simplifications and unwieldy sensor arrays required for physical modeling approaches motivate alternative methods for estimating biomechanical time-series, and especially for remote patient monitoring.

### 1.2. Regression Techniques

Regression models that capture the relationship between wearable sensor inputs and biomechanical time-series outputs may provide an opportunity to further simplify the wearable sensor system required for remote patient monitoring. These models are developed from a large number of observations through a process that may be referred to as system identification [35], function approximation [36], or machine learning [37], depending on the field. It is important to note, however, that many of the physics-based techniques also regress model parameters from a large number of observations [32], wherein that process is often referred to as calibration, and the parameters being regressed are physical constructs based on the derivation of the model from first principles (e.g., tendon slack length, muscle activation constants [24]). The current review will focus on the use of non-physical regression as a means for estimating joint, segment, and muscle kinetics and kinematics from wearable sensor data. 

### 1.3. Relevant Reviews

Techniques for estimating biomechanical time-series from wearable sensor data have been the focus of previous literature reviews. Faisal et al. (2019) recently provided a high-level overview of sensing technologies, applications of wearables in monitoring joint health, and analysis techniques [38]. Several reviews are available concerning the use of Hill-type muscle models for sEMG informed muscle force estimation which can be used to estimate kinematics via forward-dynamics [26,27,32,39]. Dowling (1997) mentions the potential use of neural networks in this context but does not review any relevant literature. Sabatini (2011) provides an overview of the use of inertial sensors for estimating segment and joint kinematics using physics-based techniques and sensor fusion algorithms [21]. Ancillao et al. (2018) review physics-based techniques for estimating ground reaction forces and moments using wearable inertial sensors [40]. While these previous reviews capture the current state of physics-based techniques well, there has not been a comprehensive review of regression techniques for estimating joint, segment, and muscle kinetics and kinematics from wearable sensor data. Schöllhorn (2004) provides a relevant review, but focuses only on neural networks and, as will be seen later, none of the articles they reviewed met the inclusion criteria outlined below and thus we also include studies using neural networks in this review [41]. Shull et al. (2014) review the applications of wearable sensors for clinical evaluation and for biofeedback, but they were only interested in gait, did not focus on the estimation technique, and none of the papers they reviewed used sEMG [42]. Caldas et al. (2017) review the application of adaptive algorithms for estimating gait phase, spatiotemporal features, and joint angles [43]. While joint angles are relevant to this review, Caldas et al. focus only on the use of inertial sensors and only mention three studies used to estimate joint angles; two of which are also included here. Finally, Ancillao et al. (2018) also reviewed machine learning techniques for estimating ground reaction forces and moments [40]. Thus, studies estimating only ground reaction forces and moments were excluded in this review.

The aim of this review was to characterize the use of regression algorithms to estimate biomechanical time-series from wearable sensor data. A secondary aim was to develop a classification method to group the prediction equations based on their technical similarities. 

## 2. Methods

### 2.1. Search Strategy

The PubMed and IEEE Xplore databases were searched for relevant articles in August 2019. Search terms were chosen to reflect the aims of the current review namely studies investigating (1) regression of (2) human biomechanical time-series using (3) wearable sensor data (see Table 1 for search terms pertaining to items 1–3). After duplicates were removed, the title and abstract of each article was screened to determine if the full text would be reviewed. 

### 2.2. Inclusion/Exclusion Criteria

Only peer-reviewed journal articles (no conference proceedings) written in English were considered. Articles were included in the review if they met all criteria within the following three categories:

(1) Sensor criteria: clear use of data for estimation from a sensor that is currently deployable as a wearable. Studies investigating model inputs dependent on virtual wearable sensor data derived from a non-wearable sensor were excluded. Studies using exoskeletons were excluded if the wearable sensor is only feasibly deployed using the exoskeleton.
(2) Prediction criteria: use of non-physical regression (not classification, regressed parameters must not be physical constructs). The estimated variable must have been a biomechanical time-series describing either the kinetics or kinematics of a joint, segment, or muscle. Studies were excluded if they estimated only grip or pinch forces unless the contact forces of each involved segment were estimated separately. Finally, studies estimating only ground reaction forces and moments were excluded as methods for this purpose have recently been reviewed [40].
(3) Validation criteria: all studies reviewed must have reported the objective (i.e., numerical) quantification of testing error using their estimation method. Studies were excluded if they report statistics for the training error only or if the only description of performance was given graphically. Studies utilizing inappropriate validation were excluded (e.g., one that could not be repeated or one using an invalid gold standard for validation).

These exclusion criteria were used for both the title/abstract screening and for full-text review. For many papers, the presence of one or several exclusion criteria was made clear via the title and/or the abstract. Therefore, these articles were removed after the title/abstract screening and were not full-text reviewed.

### 2.3. Data Analysis

All studies that met the inclusion criteria were characterized by the sample size, subject demographics (sex, health status, age), wearable sensors (type, sampling frequency), biomechanical variable estimated, tasks for which the estimation was validated, model characteristics, and estimation performance. One aim of the current review was to summarize the various estimation techniques and their performance. A detailed description of the methods and error statistics used in each study is infeasible, so we grouped prediction equations post-hoc according to a grouping method which distinguishes the different techniques for comparison (see Section 3.4). Further, we report summary statistics which summarize the overall performance (e.g., range of root mean square error across all observed tasks).

## 3. Results

**Figure 1:**
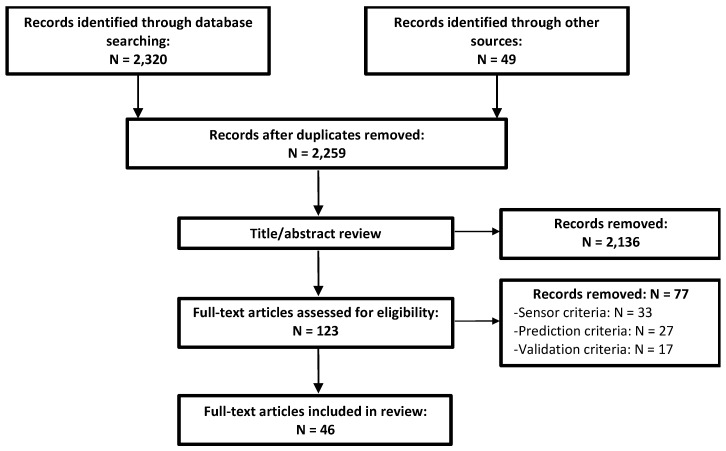
Flow chart of article selection process. Of the 123 full-text reviewed articles, 77 were removed on the basis of one or several exclusion criteria pertaining to the sensors used, the prediction approach, and/or the validation procedure. See Section 2.2 for details concerning specific exclusion criteria.

**Figure 2:**
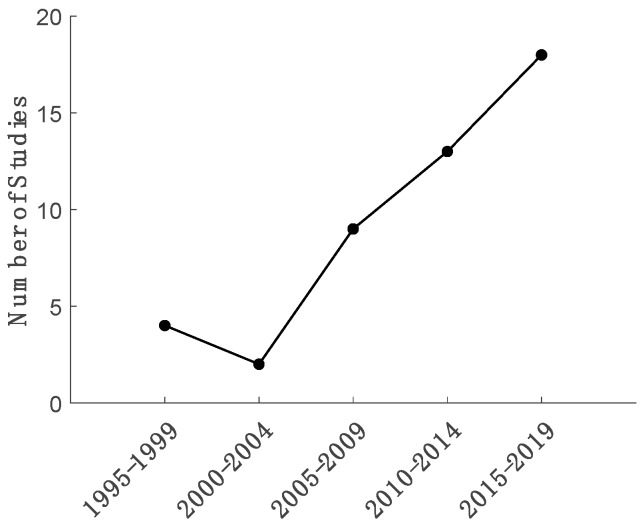
Number of articles included in the review for each five-year bin. The oldest paper included in our review was published in 1995.

A total of 46 articles met the inclusion criteria for full-text review out of 2259 distinct articles identified via database searches and from external sources (Figure 1). There was a clear increasing trend in the number of articles which met our review criteria published since the earliest identified in 1995 (Figure 2). 

### 3.1. Subject Demographics

Across all participants used for validating the regression techniques, most were unimpaired males (64%), followed by unimpaired females (29%) and impaired individuals (7%). Three studies validated their algorithm on just one person while only 11 studies validated their algorithm on a sample size of greater than 10 participants. One study [44] did not report any information concerning the subject sample (other than that they were normal subjects) and the largest sample size for which an algorithm was validated was 33 (all unimpaired, 15 female) [45]. 

### 3.2. Wearable Sensors

Surface electromyography sensors were the most popular wearable sensors used (32 studies) followed by inertial sensors (nine studies, four used magnetic/inertial measurement units, three used inertial measurement units, and two used accelerometer only) and high density sEMG (HD-sEMG) (five studies). One study used an electrogoniometer in addition to sEMG [46] and two studies used mechanomyography sensors in addition to sEMG [47,48]. Two studies used force sensitive resistors to instrument insoles [49,50] and one of these used an additional load cell over the Achilles’ tendon [50]. The average sensor sampling rate across all studies using sEMG was 2288.8 Hz (range: 500–16,000 Hz) and was 303.75 Hz across the nine studies using inertial sensors (range: 50–1500 Hz). Grid sizes for HD-sEMG included 128, 160, and 192 with an average sensor sampling rate of 1838.4 Hz (range: 1.0–2.048 kHz). 

### 3.3. Biomechanical Variables

**Figure 3:**
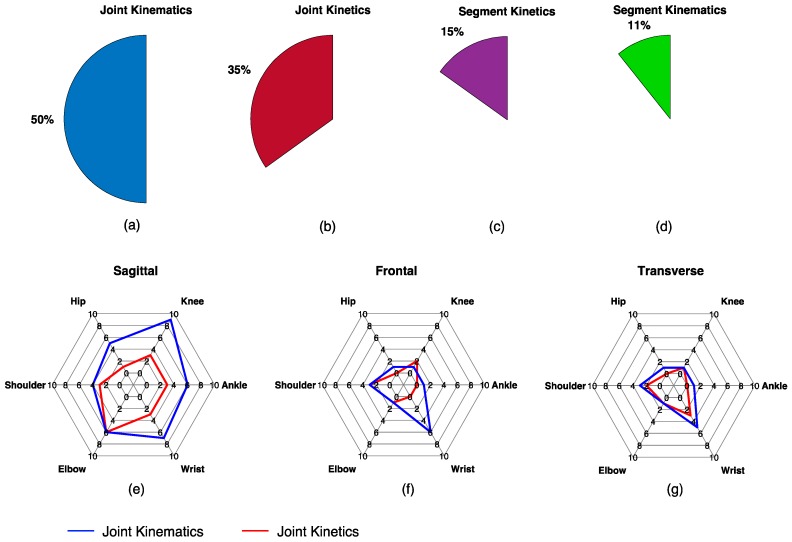
Description of the biomechanical variables estimated across all reviewed studies. The top row of figures illustrates the percentage of studies that estimated joint kinematics (**a**), joint kinetics (**b**), segment kinetics (**c**), and segment kinematics (**d**) and the bottom row of figures are radar plots illustrating the number of studies estimating the major upper and lower extremity joint kinematics (blue) and kinetics (red) in the sagittal (**e**), frontal (**f**), and transverse (**g**) planes. No studies estimated muscle forces or joint contact forces.

Across all studies, the most frequently estimated biomechanical time-series was joint kinematics (23 studies) followed by joint kinetics (16 studies), segment kinetics (seven studies), and segment kinematics (five studies) (Figure 3). Of the 16 studies estimating joint kinetics, only three estimated the intersegmental force. No studies estimated joint contact forces, individual muscle forces, or muscle kinematics. Most studies focused on joint/segment biomechanics in the sagittal plane (87%), followed by the frontal plane (46%), and transverse plane (33%) (Figure 3). Across all studies and considering the major upper and lower extremity joints, the wrist joint received the most attention (28%), followed by the knee (26%), the elbow (24%), the ankle (20%), the shoulder (15%), and the hip (13%).

### 3.4. Prediction Equations

#### 3.4.1. Prediction Equation Classification

One aim of the current review was to develop a classification method post-hoc allowing a high-level comparison of the structure of the many different prediction equations used in the reviewed papers. Note that estimation performances were not compared statistically between methods from different studies as the nature of the model validation procedures were too often different enough such that a comparison of error statistics would not be appropriate. The rest of this section describes the classification we have developed for this comparison. We feel this method best groups the reviewed papers for an insightful comparison, but it is by no means unique. The description of all techniques used in the reviewed papers according to this classification is presented in Table 2 in addition to some other study characteristics for a succinct overview of all reviewed papers. It is recommended that the description of the classification system be read first to best understand the comparison in Table 2.

We use $x\left(t\right)\in {\mathbb{R}}^{d}$ to denote the d-dimensional input used to estimate the m-dimensional output (biomechanical time-series) $y\left(t\right)\in {\mathbb{R}}^{m}$ at time t . All reviewed papers presented regression algorithms to determine the parameters of a prediction equation $f:{\mathbb{R}}^{d}\to {\mathbb{R}}^{m}$ which defines the explicit mapping x(t)→y(t). In the context of this review, the $i^{th}$ element $x_{i}\left(t\right)$ of the input x(t) may be a wearable sensor measurement after some pre-processing step (called an exogenous input) or a state variable being fed back. This state variable may be either an element $y_{i}\left(t-t_{d}\right)$ of a previous output $y\left(t-t_{d}\right)$ (i.e., at time $t-t_{d}$, $t_{d}>0$), or some other internal state (e.g., an output from a hidden neuron prior to the output layer in a neural network). All prediction equations reviewed in this paper use exogenous inputs. In this review, we use the term feedback to refer to models which also use output and/or internal state variable feedback. For example, herein Elman networks [51], long-short term memory (LSTM) neural networks [52,53], and non-linear/linear autoregressive (with exogenous inputs) models [48,54] are all considered to have a feedback structure. 

In general, an exogenous input $x_{i}\left(t\right)$ will be either the value of a sensor time-series s at time t , s(t) , or a discrete feature which describes s over some finite time interval. Note that s(t) may be the raw sensor signal itself or after some pre-processing step. For example, in this review, we classify the value of an sEMG envelope at some time instant as a time-series input, even though this value may depend on previous (or future) raw sEMG samples. Similar to system theory, we use the term dynamic to refer to models which use past exogenous inputs, for example $x_{i}\left(t-t_{d}\right)$ for $t_{d}>0$, to estimate y(t) at time t. Note the difference between what we call a dynamic structure versus a feedback structure is that dynamic refers to the use of past exogenous inputs whereas feedback refers to the use of past outputs and/or internal state variables as a part of the input. We further classify discrete exogenous inputs as time-domain (TD) if computed in the time-domain (e.g., root mean square value) and frequency-domain (FD) if computed in the frequency-domain (e.g., Fourier coefficients). We also report which studies first decomposed the sEMG into motor unit action potentials (MUAPs) from which time domain (MUAP-TD) or frequency domain (MUAP-FD) discrete features were extracted.

Previous efforts to classify prediction equations have identified two classes, (1) a mixture of linear models and (2) a weighted sum of basis functions, into which a wide range of techniques can be classified [55]. We found that all prediction equations used in the studies reviewed herein can be viewed as a weighted sum of basis functions (where the weight of any one particular basis function is not restricted to be constant as in [55]). Given this general perspective, we identified a three-class classification for grouping the techniques used in each of the 46 reviewed papers: (i) polynomial mixtures (${\mathbb{P}}^{n}$), (ii) neural networks (NN), and (iii) nonparametric regression (NP).

The ${\mathbb{P}}^{n}$ class is viewed as a special case where the basis functions are strictly nth -order polynomials, n∈ℕ. Often, models are classified as either linear or non-linear, but here we consider both first-order polynomial mixtures (n= 1) and higher order polynomial mixtures (n> 1) as sub-classes of ${\mathbb{P}}^{n}$. This is because a first-order linear model may use features which are non-linear transformations of raw sensor signals. For example, consider a model using the sEMG amplitude at time t (denoted by x(t)) for estimation. Then the prediction equation $y\left(t\right)=a_{1}x\left(t\right)+a_{2}x^{2}\left(t\right)$, for coefficients $a_{1},a_{2}\in \mathbb{R}$, may be interpreted as a linear model with two features as inputs (namely sEMG amplitude and squared sEMG amplitude) or as a 2nd order polynomial with a single input (i.e., sEMG amplitude). To improve clarity, we report both the polynomial model order and a description of the features used for estimation in Table 2. Prediction equations belonging to the ${\mathbb{P}}^{n}$ class in this review include those resulting from Gaussian mixture regression [56], lasso [57], and ridge [58] regression, and an ensemble of polynomials [58] among others.

The NN class is viewed as a special case where the basis functions are neural networks. This formulation allows for both radial basis function networks [59] and an ensemble of networks [60] as the final prediction equation.

The NP class refers to models which require access to all training data when making predictions (as defined in [36]). All NP prediction equations in this review are either linear smoothers [36,61] or (kernelized) support vector regression (SVR). Linear smoothers express the estimated output for a test input as a linear combination of all training targets. These include the prediction equations resulting from Gaussian process regression [48,62], kernel ridge regression [58], kernel smoothers [63,64], and *k*-nearest neighbors regression [65].

#### 3.4.2. Descriptive Statistics of Prediction Equations

Neural networks were the most popular model (33 studies, 72%) followed by polynomial mixtures (14 studies, 30%) and nonparametric regression (seven studies, 15%). Of the 14 polynomial mixtures, 12 were first-order (linear models) of which nine used time-series inputs. Time-series inputs were used more often (72% of studies) than discrete features (33% of studies). Across the 15 studies using discrete features as inputs, 13 contained time-domain features, three contained frequency-domain features, and three studies estimated the decomposition of the raw sEMG signals into individual MUAPs before computing discrete features. Ten studies used a dynamic structure and nine studies used a feedback structure. Seven studies used principal component analysis as an unsupervised feature reduction method. Most studies present subject-specific models (80%). No final prediction equations developed in any studies were open-sourced, but one paper [66] provided open-source code for their MUAP decomposition algorithm. Table 2 provides an overview of the prediction equations used in each study as well as a summary statistic summarizing estimation performance.

## 4. Discussion

Remote monitoring of patient segment, muscle, and joint kinematic and kinetic time-series has been established as an important component of digital health. Practical limitations in the number of sensors that can be deployed simultaneously to a given user motivate the pursuit of regression-based approaches. Thus, the primary aim of this review is to summarize relevant developments in the use of regression for estimating these biomechanical time-series. This review is timely given the increase in relevant studies since the turn of the century (Figure 2) and the limitations of other systematic reviews in the area. While many different techniques were observed since the first relevant method published in 1995, there are some common themes consistent across studies which we discuss below. Additionally, we discuss challenges concerning the practical implementation of the reviewed methods and common characteristics of the techniques that provided the best performance to provide possible directions for future work. In particular, we discuss how incorporating domain knowledge often improved performance and the implications for hybrid estimation (i.e., using both physics-based and machine learning techniques in concert). Note that our identification of techniques that may improve performance was not based on a comparison of methods between the studies reviewed herein. Instead we draw conclusions concerning techniques that led to improved performance only where those conclusions were inferred within individual studies that report an appropriate statistical comparison.

### 4.1. Overview of Techniques

Neural networks were the most popular regression model. Most incorporated a 3-layer feed forward neural network (non-recurrent, single hidden layer) [47,50,57,58,59,62,65,67,69,71,72,73,74,75,76,77,78,79,80,81,82,85] and differed based on the choice of activation function and/or number of hidden neurons. The number of hidden neurons in the NN models reviewed was usually optimized over a set of predefined values [46,47,51,54,58,62,65,69,70,72,74,75,77,78,83] but sometimes not [37,50,67,71,76,81]. Two papers considered an ensemble of networks. Koike and Kawato (1995) trained two task-specific NNs (one for postural activities and the other for dynamic) and a gating network which provided the weights for linearly combining the joint torque estimates from the two task-specific NNs [60]. Ding et al. (2017) utilized an unscented Kalman filter for combining two NNs to estimate elbow joint angle and upper arm orientation [83] wherein a recurrent NN trained using sEMG data with reduced information redundancy (using a custom reduction approach) was used to model the time-update equation and a second NN trained to estimate a redundant sEMG time-series was used as the measurement-update equation. Convolutional and long-short term memory NN (CNN and LSTM, respectively) were first used in 2018. Xia et al. (2018) found that an LSTM in series with a CNN (C-LSTM) outperformed a CNN alone for estimating hand position during general open-chain tasks [52]. Likewise, Xu et al. (2018) found that C-LSTM outperformed LSTM alone which outperformed CNN alone (nRMSE: 8.67%, 9.07%, and 12.13% respectively) for estimating contact forces at the distal forearm and was one of the few studies to use a leave-one-subject-out validation approach [53]. 

Polynomial mixtures were the next most popular model and of these, first order polynomials were most common. Consideration of simple linear models is motivated by an observed relationship between sEMG amplitude and muscle force, especially at lower force levels. However, to increase muscle force, additional motor units are recruited and/or stimulation frequency increases which along with heterogenous activation within a muscle and load sharing between muscles makes this relationship non-linear [27,32]. Some reviewed papers compared linear models (${\mathbb{P}}^{1}$) to both neural networks [57,58,74] and nonparametric regression [48,57,58]. Although between model comparisons varied and two of these four studies only considered isometric tasks [57,74], the NN and NP performances were no different than those from linear models. Comparisons have also been made between first order and higher order polynomial mixtures. It was shown in [68] that linear models performed equally as well as second order models for estimating lumbo-sacral joint torque using sEMG and Clancy et al. (2006) show that superior sEMG amplitude estimation techniques (e.g., whitening, multi-channel) can improve linear models [35]. Alternatively, Clancy et al. (2012) show that 2nd or 3rd order polynomials outperformed 1st and 4th order models (with regularization and optimal dynamic orders) for estimating isometric elbow joint torque using sEMG inputs [45]. A few studies considered an ensemble of polynomials. Michieletto et al. (2016) used Gaussian mixture regression, which can be shown to be a linear mixture [55], to estimate knee flexion/extension angle using sEMG inputs [56]. Hahne et al. (2014) used degree-of-freedom-specific linear models to estimate wrist joint angle and linearly combined their estimates using weights determined by a logistic regression model trained to classify the degree-of-freedom of the movement (the weights were the posterior class probabilities) [58].

Nonparametric regression was used least frequently. This may be due to the amount of data necessary to compute an estimate given the nonparametric models used in the reviewed studies (although reduction methods exist [36]). While this may be prohibitive for real-time applications (e.g., for prosthetic control [58]) it may still be a feasible method for remote patient monitoring applications where data can be stored locally during the day and processed at a later time. Linear smoothers were the most popular nonparametric regression. The first study to use nonparametric regression in the proposed context was in 2008 where the Nadaraya-Watson estimator, a kernel smoothing technique, was used to estimate lower extremity joint angles using IMU data [63]. Goulermas et al. (2008) built upon this model by incorporating an additional term in the Gaussian kernel intended to accentuate or attenuate a training target’s contribution to the final estimate according to a custom pattern similarity index [64]. Several papers noted the advantage of nonparametric regression for small training sets. For example, Ngeo et al. (2014) show Gaussian process regression outperformed a neural network in estimating finger joint angles using sEMG data, especially for smaller data sets [62]. Similarly, Hahne et al. (2014) found that kernel ridge regression outperformed a neural network for both a reduced training set and when reducing the number of sEMG channels of a high-density array (from 192 to 12–16) [58].

### 4.2. Concerns for Practical Implementation

Remote patient monitoring and myoelectric prosthetic control were the two most common applications used to motivate the many different techniques reviewed which indicates that eventual users of these systems are expected to present with clinical impairment. However, our results show that most studies do not validate their estimation techniques on impaired individuals. Evaluating algorithm performance on unimpaired populations is certainly useful for algorithm development as it reduces extraneous variables and simplifies study recruitment and retention efforts. Nevertheless, these algorithms need to be deployed to impaired populations and, while some studies present improved or equal performance for impaired individuals, many show that performance decreases. Thus, caution should be taken when considering how well a technique will work when deployed for a population on which it has not been validated. This clearly applies for a model trained on healthy participants but deployed to participants with impairment (though in some cases the drop in performance is minimal [90]). However, one also cannot assume that a model trained and tested on impaired participants will have identical performance characteristics as the same model trained and tested on healthy participants.

In addition to generalizing performance across populations, more research is needed to better understand how these regression models generalize across individuals and tasks. The majority of studies (80%) developed subject-specific models and only 33% of studies explored task extrapolation. The latter may be less of a barrier to implementation since in practice task identification will likely be a part of the pipeline for automated analysis [91], in which case highly accurate activity classification models are required [92]. Thus, task specific models could be selected following task identification. However, given the approaches reviewed herein, subject-specific models require every user to be observed in-lab for model training. Further, the observation sets for model training must be broad enough in scope (e.g., multi-speed, multi-load) so that they can be confidently applied for estimation in unconstrained environments. These requirements substantially limit the scalability of these solutions for remote patient monitoring. Subject-general models may be one of the more difficult challenges to overcome in the future as they appear to frequently result in performance decreases [59,63,64,85]. Intuitively, this may indicate that current regression models are learning person-specific patterns as opposed to generalizable phenomena. This may be a result of the small sample sizes used for model training in many of the reviewed studies. To fully realize the potential of regression techniques for estimating biomechanical time-series, future work should incorporate observations from impaired populations in their training and validation sets and larger sample sizes to foster learning of generalizable phenomena.

The clinical utility of the reviewed estimation techniques is largely driven by the estimated biomechanical variables. This review found no relevant studies which estimated muscle or joint contact forces. This is likely due to the fact that direct measurement of these variables is substantially more invasive than joint or segment mechanics. Nevertheless, indirect muscle and joint contact force estimates enabled by traditional laboratory-based gait analysis can be informative clinically [18,19]. Thus, models trained using these indirect estimates as training targets may be useful for estimating muscle and joint contact forces in remote environments. Further, future research should investigate the estimation of frontal and transverse plane joint mechanics. Specifically, frontal plane joint moment may be especially useful in monitoring patients at risk of developing knee osteoarthritis and remote observation of these mechanics may provide clinical endpoints to evaluate intervention efficacy or inform rehabilitation decision making [13,93]. There is room for improvement in this area as only one study [49] reports the estimation of non-sagittal plane moment of any lower extremity joint (frontal plane knee joint moment during walking), and performance was inferior to sagittal plane estimates achieving normalized root mean squared error of 16.4 ± 5.7% (vs. 10.7 ± 5.3% for sagittal plane moments) in healthy subjects. 

Deployment of many of the reviewed techniques is further complicated by hardware limitations. Of particular concern are the battery capacity and memory constraints of current wearables. Of the more popular wearable sensors, gyroscopes are notorious for limiting long-term capture due to their power requirements and would thus limit immediate application of several methods reviewed [37,63,64,65,76,81,85]. Alternatively, accelerometers and sEMG are able to provide continuous recording for at least 24-h with current battery technology. The use of sEMG for remote monitoring is less common than accelerometry and has been used primarily for quantifying indices of physical activity [94,95,96,97]. Recent efforts have estimated muscle activation time-series during walking using methods similar to those used to estimate muscle force using Hill-type muscle models [8,91,98]. This pre-processing step was used by several reviewed papers suggesting they may be practically deployed. However, the sEMG sampling frequency used in many of the reviewed studies (500 Hz to 16 kHz) was much higher than what has been used for remote monitoring (10–250 Hz). It is currently unknown to what extent estimation performance is influenced by sEMG sampling frequency. Future research should explore these limitations in search of hardware and algorithmic solutions that are practically deployable for remote patient monitoring.

An additional practical concern is the number of wearable sensors required for the reviewed algorithms. Several studies considered the effect of reducing the number of sensors on estimation performance. Clancy et al. (2017) present a backward stepwise selection method for reducing the number of necessary sensors [84]. They show that additional sensors beyond four (up to 16) provided no statistically significant advantage for estimating degree-of-freedom-specific wrist joint kinetics. This reduction method was later used by Dai et al. (2019) for a similar application where the reduction approach generally outperformed pre-selected sensor locations [88]. Dai and Hu (2019) present a method for reducing a high-density grid of 160 sEMG electrodes down to an 8 × 8 grid, however, the 8 × 8 subset was finger specific (for estimating finger kinematics) [87]. Future work in the development of regression approaches for estimating biomechanical time-series should incorporate analysis of the effect of reducing instrumentation complexity (i.e., reducing the number and types of sensors required) on estimation performance. 

Finally, only one study provided open-source code for any part of their methodology [66]. The code was for performing the MUAP decomposition of the raw sEMG signals and not the actual regression model. Open-sourcing subject-general models will allow non-specialized research teams without expertise in engineering or computer science to utilize these methods for clinical purposes. Further, it will allow 3rd party validation; a necessary component prior to practical deployment and to promote confidence from the public in the clinical utility of these tools. Open-source data as well as open-source code in future studies would help speed the pace of development of these techniques.

### 4.3. Incorporating Domain Knowledge

While we excluded physics-based techniques from the current review, several papers incorporated domain knowledge into their approach (e.g., muscle and neural physiology, rigid body dynamics) which was often reported to improve performance. For example, Koike and Kawato (1995) incorporated feedback of joint angular position and velocity specifically on the basis of the well known force-length and force-velocity properties of muscle [60]. Further, pre-processing of the raw sEMG signals to optimally estimate sEMG amplitude was often motivated by an understanding of muscle activation dynamics. State-of-the art estimation incorporates signal whitening and the use of multiple channels (multiple sensors per muscle) [32,35,99]. These techniques have been shown to improve estimation performance compared to other methods [35,45]. Most papers used the standard highpass filter, rectify, lowpass filter processing to estimate sEMG amplitudes and a broad range of lowpass filter cutoff frequencies were used [15,46,48,53,54,56,57,62,67,68,69,72,76,86,88]. In addition to enveloping techniques, some incorporate the fact that the observed sEMG is the superposition of many MUAPs. Three studies (all since 2018) computed discrete features as model inputs after first performing MUAP decomposition (Table 2). Given their results, Dai and Hu (2019) recommend the MUAP decomposition over standard enveloping techniques [87]. Sun et al. (2018) identified shape-based clusters (K-means, 5≤K≤20) of MUAPs extracted from the biceps brachii sEMG and suggest the different clusters represent different motor units [66]. The final estimation can be seen as a scaling of a single feature related to the number of activated motor units which they use to represent firing rate (see Equation (10) in [66]). Thus, the pre-processing of the raw sEMG signal, to estimate both sEMG amplitude and MUAPs, based on its physiological origin [32,99] may have contributed to improved estimation performance. An electromechanical delay (delayed increase in muscle force following neural excitation) is also known to characterize muscle contraction dynamics [32]. This phenomenon may provide a physiological justification for the improvements in performance associated with the use of a dynamic model structure allowing previous sEMG values to have lasting effects on the estimated output. Total delay was sometimes optimized using a grid search (625–875 ms [69], 50–150 ms [54]) and sometimes not (130 ms [68], 0.5 ms [44], 488.3 ms [88]). Clancy et al. (2006) found that performance increased with greater total time delay up to about 10 or 15 samples (i.e., 244.1 or 366.2 ms) [35]. Likewise, Clancy et al. (2012) tried between 1 and 30 sample delays and found that lesser time delays (namely total delay <5 samples or 122.1 ms) resulted in poorer performance [45]. Overly large delays also resulted in poor performance, especially for high polynomial orders which they attribute to overfitting. The best total delays (439.5–683.ms) were dependent on polynomial order and the regularization method. Ngeo et al. (2014) modeled the sEMG to activation dynamics using the method described in [100] and optimized the electromechanical delay. Optimal values were person-specific (between 39.6–75 ms) and they show that incorporating electromechanical delay into their activation model improved performance compared to neglecting it [62]. Some of the optimal delays reported in the reviewed studies are larger than what is reported elsewhere in the literature (30–150 ms) [32]. One explanation may be that in addition to the delayed effect of neural excitation, more information concerning the sEMG time-history could help a regression algorithm capture some sub-task related neural control pattern which may be inferred from a sufficiently large (i.e., >150 ms) window of time. The muscle synergy hypothesis may provide a physiological basis for expecting said pattern to exist [101]. This concept was mentioned in several reviewed papers and thus we pay it special attention next.

#### 4.3.1. Reference to Muscle Synergies

Several papers referred to the muscle synergy hypothesis in the development of their models and in the discussion of its performance. The muscle synergy hypothesis provides a potential explanation of how the central nervous system accommodates redundancy in motor control [102]. The theory suggests that the activation time-series of a given muscle is a linear combination of a small set of basis waveforms. Non-negative matrix factorization (NMF) is an algorithm commonly used in muscle synergy analysis to optimally determine the basis functions and the coefficients for linear combination given a set of muscle sEMG or activation time-series [101,102,103]. Jiang et al. (2009) used these techniques directly in their estimation and show that for estimating contact forces at the hand, their method using NMF is nearly unsupervised in that target force values are not needed and is only supervised in the sense that the degree-of-freedom must be known for model training [74]. Others have referred to muscle synergies as a possible explanation for the observed accuracy of some regression techniques [35,69,70,82]. The synergy hypothesis indicates that the activity of all muscles contributing to a given joint torque may be approximated given a common and observable subset of sEMG observations. While the estimation of muscle activation time-series was not included in the current review, we note that Bianco et al. (2018) explored the possibility of estimating unmeasured muscle activations from sEMG time-series measured from eight different muscles using the traditional linear combination of basis waveforms formulation of muscle synergies [104]. To the authors’ knowledge, no studies have regressed unmeasured muscle activations using a reduced number of wearable sensors. In this formulation, the function being identified in the regression would effectively model the synergistic relationship between muscles. Such an approach might enable estimated activations to inform a complete set of Hill-type muscle models crossing the joint of interest to estimate muscle force. Wang and Buchannan (2002) tried a similar approach wherein a neural network was trained to learn the muscle activation dynamics (intramuscular EMG to muscle activation model) using estimated torque error to drive parameter adaptation in the learning process [105]. However, they estimated activations only for those muscles with measured intramuscular EMG. Thus, advances in modeling the observed synergistic behavior of muscle activations may prove useful for improving estimation of biomechanical time-series with a minimal number of wearable sensors. 

The muscle synergy hypothesis suggests that an observed set of muscle activation or sEMG time-series carries redundant information and can be explained by a lower dimensional structure (e.g., less than the number of sensors available). Regularization is a common technique in machine learning used to reduce model complexity and prevent overfitting, usually at the expense of training error. Reducing the number of inputs by removing redundant information also reduces model complexity and the muscle synergy hypothesis may provide a physiological basis for this phenomenon. Clancy et al. (2012) compared ridge regression to their pseudo-inverse based regularization wherein the reciprocals of singular values below some threshold were replaced with zero [45]. The best ridge regression results were similar to the pseudo-inverse regularization, however, optimal fits were less sensitive to changes in pseudo-inverse tolerances near the optimum than they were to changes in the ridge penalty hyperparameter suggesting the pseudo-inverse technique may be easier to tune. This technique, also used in [84,88], along with self-organizing maps [73] and principal component analysis [53,58,75,78,82,86,89] are examples of unsupervised feature reduction techniques. Chen et al. (2018) found that using a deep belief network to reduce 10 inputs to three outperformed the PCA approach for the same dimensionality reduction task [86]. This might be considered a supervised dimensionality reduction (as would lasso regression [57]) as the determination of the weights in the hidden neurons of the deep belief network are optimized so that the final output best approximates the training set targets. Thus, although feature reduction is common in machine learning for improving generalizability, it may be further justified on a physiological basis given the assumption that a lower dimensional structure of the inputs exists.

#### 4.3.2. Towards a Hybrid Approach

A general conclusion from these observations is that clever incorporation of domain knowledge in regression techniques may improve performance. In the papers we reviewed, this was mostly by way of sensor signal pre-processing, feature engineering, and model structure (e.g., feedback or dynamic). Incorporation of domain knowledge in regression has been suggested for other biomechanics applications [106], and as shown in [36], a good understanding of system dynamics can directly inform kernel structure in Gaussian process regression. For these reasons, hybrid methods using both physics-based and machine learning techniques in concert are being proposed in other fields including climate sciences [107], GPS-inertial navigation [108], and general chaotic processes [109]. As noted in a recent editorial [110] concerning climate modeling, “The hybrid approach makes the most of well-understood physical principles such as fluid dynamics, incorporating deep learning where physical processes cannot yet be adequately resolved.” The general approach observed in many of these techniques are generalizable and applicable beyond specific scientific disciplines and thus may prove beneficial for remote patient monitoring. One approach might be to regress an unobserved internal state for which the physical relationship with observed measurements is either not well understood or not fully informed (e.g., not enough sensors) and then to drive a physical model using the estimated internal state variable. For example, this was done in [105] where the authors’ chose to model muscle activation dynamics using a neural network since they determined these dynamics to be the least well understood. A second approach might be the fusion of a regression estimate and a physical model estimate. Along these lines, if uncertainties are modeled, the parameters of the regression (or the physical model) may be adapted in real-time. Gui et al. (2019) use a similar approach to remove the need to calibrate an EMG-torque model [111]. In the proposed context this could be especially useful as it may be interpreted as real-time subject specification from a general model. Further, it may enable the adaptation of a model to time-varying signal characteristics (e.g., due to electrode displacement, changes in skin conductivity, specific spatial position of inertial sensors) which may negatively impact estimation [57]. Future developments in hybrid methods that take advantage of the strengths of both physical models and machine learning may help realize the maximum potential of remote patient monitoring.

## 5. Conclusions

Regression techniques present an alternative approach to physical models for estimating biomechanical time-series using wearable sensor data. These methods could be transformative for personalizing healthcare interventions as they allow the monitoring of a patient’s biomechanics continuously and in unconstrained environments. The aim of this review was to summarize relevant regression techniques in this context to imply directions for future research concerning practical implementation and improving estimation performance. Several reviewed studies found that incorporating some form of domain knowledge resulted in better estimation accuracy. Advances in this area along with open-source algorithms, validation in impaired populations, and consideration of practical hardware limitations (e.g., battery capacity and memory) may expedite future developments to make clinical implementation a reality. In summary, future work should consider the following:

▪ Development of methods using hardware specifications that can be implemented remotely and for a full 24-h capture.
▪ Development of subject-general models or real-time calibration.
▪ Development of hybrid machine learning and physics-based estimation.
▪ Open-source algorithms.
▪ Development of regression models for estimating muscle forces and joint contact forces.
▪ Validation of models on impaired populations.

## Figures and Tables

**Table 1:** Search terms and the item pertaining to this review that they reflect.

Review Relevant Item	Search Terms
Regression	regress* OR “machine learning” OR “artificial intelligence” OR “statistical learning” OR “supervised learning” OR “unsupervised learning” OR “neural network” OR perceptron OR “support vector” OR “gaussian process”
	AND
Biomechanical Time-Series	joint OR limb OR segment OR ankle OR knee OR hip OR wrist OR elbow OR shoulder OR muscle AND angle OR velocity OR acceleration OR moment OR torque OR force OR kinematic* OR kinetic* OR biomechanics OR mechanics OR dynamics
	AND
Wearable Sensors	wearable OR accelerometer OR gyroscope OR electromyo* OR EMG OR sEMG OR “inertial sensor” OR “inertial measurement unit” OR IMU OR insole OR goniometer

**Table 2:** Overview of the 46 reviewed papers.

Reference (Year)	$\mathrm{Sensors}\;(f_{s},\;\mathrm{Max}\;\mathrm{Number})$	Variable (Location): Plane(s)	Tasks	Inputs	Model	Performance Summary
Koike and Kawato [60] (1995)	sEMG (2 kHz, 10)	τ (elbow): S τ (shoulder): F	ISO, OC	TS	NN (FB, dyn)	CD: 0.89
Suryanarayanan et al. [44] (1996)	sEMG (2 kHz, 1)	θ (elbow): S	OC	TS	NN (dyn)	RMSE ≤ 15%
Shih and Patterson [67] (1997)	sEMG (900 Hz, 4)	τ (elbow): S τ (wrist): S τ (shoulder): S θ (elbow): S θ (wrist): S θ (shoulder): S	WCP	TS	NN	RMSE: 0.67–5.76 Nm, **0.64–5.62** Nm RMSE: 4.78–13.76°, **4.73–14.33**°
van Dieën and Visser [68] (1999)	sEMG (600 Hz, 6)	τ (lumbo-sacral): S	ISO, LOC	TS	$\;\;{\mathbb{P}}^{1}$ (dyn)	RMSE: 26–54 Nm, *49–160* Nm
Au and Kirsch [69] (2000)	sEMG (500 Hz, 6)	θ (shoulder): S, F, T θ (elbow): S $\dot{\theta }$ (shoulder): S, F, T $\dot{\theta }$ (elbow): S $\ddot{\theta }$ (shoulder): S, F, T $\ddot{\theta }$ (elbow): S	OC, LOC	TS	NN (dyn)	RMSE: 14.2–19.6° RMSE: 8–17.2° (impaired subjects)
Dipietro et al. [70] (2003)	sEMG (1 kHz, 5)	p (hand): T	OC	TS	NN (FB)	RMSE: 7.3–11.5%
Song and Tong [46] (2005)	sEMG (1 kHz, 3) goni (1 kHz, 2)	τ (elbow): S	LOC	TS	NN (FB)	nRMSE: 4.53–8.45% nRMSE: 10.56–16.20% (sEMG only)
Clancy et al. [35] (2006)	sEMG (4096 Hz, 8)	τ (elbow): S	ISO	TS	$\;\;{\mathbb{P}}^{1}$ (dyn)	MAE: 7.3%
Došen and Popovič [71] (2008)	2D ACC (200 Hz, 4)	θ (ankle): S θ (knee): S θ (hip): S $\ddot{p}$ (hip joint center): S	MSW	TS	NN (dyn)	RMSE: 1.19–3.60°, *1.18–2.62*° RMSE: 0.26–0.39 m/s^2^, *0.29–0.46* m/s^2^ CC (θ): 0.97–0.998, *0.97–0.998* CC ($\ddot{p}\;$): 0.96–0.99, *0.91–0.99*
Findlow et al. [63] (2008)	IMU (100 Hz, 4)	θ (ankle): S θ (knee): S θ (hip): S	Normal Walk	TS	NP (KS)	MAE: 1.69–2.30°, **4.91–9.06°** MAE: 1.78–5.32° (reduced sensor array) CC: 0.93–0.99, **0.70–0.89** CC: 0.87–0.99 (reduced sensor array)
Goulermas et al. [64] (2008)	IMU (--, 4)	θ (ankle): S θ (knee): S θ (hip): S	MSW	TS	NP (KS)	CC: 0.97, *0.96*, **0.83**
Hahn and O’Keefe [72] (2008)	sEMG (1 kHz, 7)	τ (ankle): S τ (knee): S τ (hip): S	Normal Walk	TS	NN	CD: 0.54–0.84 (sEMG only) CD: 0.77–0.92 (sEMG with demographics & anthropometrics)
Mijovic et al. [59] (2008)	2D ACC (50 Hz, 2)	$\;\;\ddot{\theta }$ (forearm): S	OC	TS	NN (RBF)	CD: 0.841–0.998, **0.75–0.99, *0.03–0.88***
Delis et al. [73] (2009)	sEMG (1744.25 Hz, 2)	θ (knee): S	Normal Walk	DISC (TD)	NN (SOM)	CC: 0.59–0.84
Jiang et al. [74] (2009)	sEMG (1 kHz, 8)	CF (hand)	ISO	DISC (TD)	(1) NN (2) ${\mathbb{P}}^{1}\;\;$	(1) CD: 0.86 (2) CD: 0.78
Youn and Kim [47] (2010)	sEMG (1 kHz, 2) MMG (1 kHz, 2)	CF (hand)	ISO	DISC (TD)	NN	nRMSE ≤ *16% (MMG only)* nRMSE ≤ *13% (sEMG only)* nRMSE ≤ *10% (sEMG + MMG)*
Ziai and Menon [57] (2011)	sEMG (1 kHz, 8)	τ (wrist): S	ISO	TS	(1) ${\mathbb{P}}^{1}\;\;$ (2) ${\mathbb{P}}^{1}$ (lasso) (3) ${\mathbb{P}}^{1}$ (LWPR) (4) NP (SVR) (5) NN (2L)	(1) nRMSE: 2.88% (2) nRMSE: 2.83% (3) nRMSE: 3.03% (4) nRMSE: 2.85% (5) nRMSE: 2.82%
Nielsen et al. [75] (2011)	sEMG (1024 Hz, 7)	CF (hand)	ISO	DISC (TD)	NN	RMSE: 0.16 N RMSE: 0.10 N (impaired subjects) CD: 0.93 CD: 0.82 (impaired subjects)
de Vries et al. [76] (2012)	MIMU (50 Hz, 4) sEMG (1 kHz, 13)	ISF (SC): S, F, T ISF (AC): S, F, T ISF (shoulder): S, F, T ISF (elbow): S, F, T	LOC, ADL	TS	NN	nRMSE: *7–17*%
Jiang et al. [77] (2012)	sEMG (2048 Hz, 7)	θ (wrist): S, F, T	OC	DISC (TD)	NN	CD: 0.74–0.78
Muceli and Farina [78] (2012)	HD-sEMG 128 (2048 Hz, 2)	θ (wrist): S, F, T	OC	TS	NN	CD: 0.79–0.89
Clancy et al. [45] (2012)	sEMG (4096 Hz, 2)	τ (elbow): S	ISO	TS	$\;\;{\mathbb{P}}^{1}$, ${\mathbb{P}}^{2}$, ${\mathbb{P}}^{3}$, ${\mathbb{P}}^{4}$ (dyn)	nMAE: 4.65–6.38% nMAE: 5.55–7.97% (reduced training set)
Howell et al. [49] (2013)	FSR (118 Hz, 12)	τ (ankle): S τ (knee): S, F	Normal Walk	TS	$\;\;{\mathbb{P}}^{1}\;\;$	nRMSE: 5.9–17.1% CC: 0.82–0.97
Kamavuako et al. [79] (2013)	sEMG (10 kHz, 6)	τ (wrist): S, T	ISO	DISC (TD)	NN	nRMSE: 6.1–13.5% CD: 0.87–0.91
Jiang et al. [80] (2013)	sEMG (2048 Hz, 7)	θ (wrist): S, F, T	OC	DISC (TD)	NN	CD: 0.63–0.86, *0.34–0.74* CD: 0.61–0.77, *0.46–0.59* (impaired subjects)
Farmer et al. [54] (2014)	sEMG (1 kHz, 4)	θ (ankle): S	Normal Walk	TS	NN (FB, dyn)	RMSE: 1.2–5.4°
Ngeo et al. [62] (2014)	sEMG (2 kHz, 8)	θ (MCPs): S	OC	TS DISC (TD)	(1) NN (dyn) (2) NP (GPR, dyn)	(1) CC: 0.71 (TS inputs only) (2) CC: 0.84 (TS inputs only)
Hahne et al. [58] (2014)	HD-sEMG 192 (2048 Hz, 1)	θ (wrist): S, F	OC	DISC (TD)	(1) ${\mathbb{P}}^{1}$ (ridge) (2) ${\mathbb{P}}^{1}\;\;$ (3) NN (4) NP (KRR)	(4) CD: 0.8 (reduced sensor array) CD: 0.8–0.9 (range across all models)
Jacobs and Ferris [50] (2015)	FSR (1 kHz, 8) Load Cell (1 kHz, 1)	τ (ankle): S	MSW, Calf Raises	TS	NN	nRMSE: 7.04–13.78% nRMSE: 8.72–16.52% (FSR only) nRMSE: 20.47–46.02% (Load Cell only)
de Vries et al. [81] (2016)	MIMU (50 Hz, 4) sEMG (1 kHz, 13)	ISF (shoulder): S, F, T	LOC, ADL	TS	NN	nSEM: *4–1%* nSEM: *3–21*% (reduced sensor array)
Wouda et al. [65] (2016)	MIMU (240 Hz, 5)	θ (ankle): S, F, T θ (knee): S, F, T θ (hip): S, F, T θ (shoulder): S, F, T θ (elbow): S, F, T θ (wrist): S, F, T θ (spine): S, F, T	OC, ADL, MSW, MSR, sport	TS	(1) NN (2) NP (*k-*NN)	(1) Mean Error: **7°** (2) Mean Error: **8°**
Michieletto et al. [56] (2016)	sEMG (1 kHz, 8)	θ (knee): S	Seated Kick	TS	$\;\;{\mathbb{P}}^{1}$ (GMR)	Custom error statistic (see paper)
Xiloyannis et al. [48] (2017)	sEMG (--, 5) MMG (--, 5)	$\;\;\dot{\theta }$ (MCPs): S	OC, ADL, ISO	TS	$(1)\;{\mathbb{P}}^{1}$ (FB) (2) NP (GPR, FB)	(1) CC: *0.54* (2) CC: 0.79, *0.62*, 0.67 (sEMG only)
Zhang et al. [82] (2017)	sEMG (1 kHz, 8)	θ (shoulder): S, F, T θ (elbow): S	OC	DISC (TD)	NN	CD: 0.90–0.91, *0.86–0.87*
Ding et al. [83] (2017)	sEMG (2 kHz, 8)	θ (elbow): S θ (humerus): S, F, T	OC, ADL	TS	(1) NN (2) NN (FB) (3) NN (FB, UKF)	(1) RMSE: *11–14°*, CC: *0.88–0.90* (2) RMSE: *11–15°*, CC: *0.87–0.89* (3) RMSE: *7–9°*, CC: *0.95–0.96*
Clancy et al. [84] (2017)	sEMG (2048 Hz, 16)	CF (hand): S, F τ (wrist): T	ISO	TS	$\;\;{\mathbb{P}}^{1}\;\;$	RMSE: 6.7–10.6%, *11.0–15.7* (4 sensors)
Xia et al. [52] (2018)	sEMG (2 kHz, 5)	p (hand): S, F, T	OC	DISC (FD) DISC (TD)	1) NN (CNN) 2) NN (C-LSTM, FB)	(1) CD: 0.78 (2) CD: 0.90
Wouda et al. [85] (2018)	MIMU (240 Hz, 3)	θ (knee): S	MSR	TS	NN	RMSE: *2.27–8.41°*, **6.29–25.05°** CC: *0.98–0.99*, **0.77–0.99**
Sun et al. [66] (2018)	sEMG (16 kHz, 1)	CF (forearm)	ISO	DISC (MUAP-TD)	$\;\;{\mathbb{P}}^{1}\;\;$	CD: **0.72–0.89**
Chen et al. [86] (2018)	sEMG (1.2 kHz, 10)	θ (ankle): S θ (knee): S θ (hip): S	MSW	TS	NN (DBN)	RMSE: 2.45–3.96° CC: 0.95–0.97
Xu et al. [53] (2018)	HD-sEMG 128 (1 kHz, 1)	CF (forearm)	ISO	TS	(1) NN (CNN) (2) NN (LSTM, FB) (3) NN (C-LSTM, FB)	(1) nRMSE: *7.33–10.93%* (2) nRMSE: *6.16–9.33%* (3) nRMSE: *5.95–9.74%*
Wang et al. [51] (2019)	sEMG (1.6 kHz, 5)	θ (knee): S	LOC	DISC (FD)	NN (FB)	nRMSE: 3.55–5.13%
Dai and Hu [87] (2019)	HD-sEMG 160 (2048 Hz, 1)	θ (MCPs): S	OC	TS, DISC (MUAP-FD)	$\;\;{\mathbb{P}}^{2}\;\;$	CD: 0.66–0.81 (TS inputs) CD: 0.69–0.86 (MUAP-FD inputs)
Dai et al. [88] (2019)	sEMG (2048 Hz, 16)	CF (hand): S, F τ (wrist): T	ISO	TS	$\;\;{\mathbb{P}}^{1}$ (dyn)	RMSE: 7.3–9.2%, *11.5–13.0%* (4 sensors)
Kapelner et al. [89] (2019)	HD-sEMG 192 (2048 Hz, 3)	θ (wrist): S, F, T	OC	DISC (TD, MUAP-TD)	$\;\;{\mathbb{P}}^{1}\;\;$	CD: 0.77 (MUAP-TD inputs) CD: 0.70 (TD inputs)
Stetter et al. [37] (2019)	IMU (1.5 kHz, 2)	ISF (knee): S, F, T	MSW, MSR, sport	TS	NN (2L)	nRMSE: **14.2–45.9%** CC: **0.25–0.94**

**Sensors:**$f_{s}$: sampling frequency (—indicates $f_{s}$ not reported), ACC: accelerometer; IMU: inertial measurement unit (accelerometer + gyroscope); MIMU: IMU with magnetometer, HD-sEMG *N*: high density grid of *N* surface electromyography electrodes, FSR: force sensitive resistors (instrumented insole); MMG: mechanomyography; goni: electrogoniometer; **Variables:** τ: net joint (muscle) moment; $\theta ,\dot{\theta },\ddot{\theta }$: joint/segment angular position, velocity, acceleration; $p,\dot{p},\ddot{p}$: segment position, velocity, acceleration; ISF: joint intersegmental force; CF: joint/segment contact force, AC: acromio-clavicular joint, SC: sterno-clavicular joint, MCPs: one or several of the metacarpophalangeal joints; **Tasks:** ISO: isometric; OC, LOC: open-chain, loaded open-chain; MSW: multi-speed walking; ADL: activities of daily living (brushing teeth, drinking, etc.); MSR: multi-speed running; sport: sport related movements (e.g., jumping, kicking, throwing); **Inputs:** TS: time-series; DISC: discrete; TD, FD: time-domain, frequency domain; MUAP: sEMG data were first decomposed into motor unit action potentials from which discrete features were extracted; **Model:** FB: model exhibits output and/or internal state variable feedback (includes autoregression); dyn: dynamic (dependent on previous inputs); ${\mathbb{P}}^{n}$: mixture of n-th order polynomials; GMR: Gaussian mixture regression; NN: neural network; RBFN: radial basis function network; SOM: self-organizing map; DBN: deep belief network; NP: nonparametric regression; KS: kernel smoother; GPR: Gaussian process regression; SVR: support vector regression; KRR: kernel ridge regression; *k*-NN: *k* nearest neighbors regression; UKF: unscented Kalman filter; CNN: convolutional neural network, LSTM: long-short term memory network, C-LSTM: CNN in series with LSTM; 2L: two hidden layers; **Performance Summary:** RMSE: root mean square error; nRMSE: normalized RMSE (e.g., RMSE in physical units normalized by maximum); MAE: mean absolute error; nMAE: normalized mean absolute error (see nRMSE); nSEM: normalized standard error of measurement; CC: correlation coefficient; CD: coefficient of determination; *italic performance metrics indicate results for task extrapolation (e.g., trained on normal walking data, tested on fast walking data*), **bold performance metrics indicate results for subject extrapolation (all data in the test set were associated with different subjects than were data in the training set)**.
